# Supplementary material for: Cold Exposure Rejuvenates the Metabolic Phenotype of Panx1−/− Mice
Source: Biomolecules. 2024 Aug 25;14(9):1058. doi: 10.3390/biom14091058 (PMC11430693; doi:10.3390/biom14091058)
Supplement: Supplementary file 1 [file biomolecules-14-01058-s001.zip › Legend to supplementary figures_biomolecules3111710_proofs.pdf]

Legend to supplementary figures:

Supplementary figure S1: 14 weeks-old *Panx1$^{-/-}$* mice have increased activity and anaerobic metabolism. (A) Body weight of WT (in black) and *Panx1$^{-/-}$* (in red) mice was measured from the age of 6 to 14 weeks. **(B)** Ambulatory, **(C)** fine and **(D)** total activity as well as **(H)** the VO_2 and **(I)** the VCO_2 were measured by indirect calorimetry on 14 weeks-old wild-type (WT; in black) and *Panx1$^{-/-}$* mice (in red). Mice were housed by two per cage. Each cage was considered as one pooled sample (N=3). **(E)** Representative images of small intestines from WT and *Panx1$^{-/-}$* mice and quantification of small intestine **(F)** and colon **(G)** lengths. Data are shown as individual data points, except for time lines where the number of mice is specified, and expressed as mean ± SEM. * $p \leq 0.05$, and $^{‡} p \leq 0.001$.

Supplementary figure S2: The activity of 20 weeks-old *Panx1$^{-/-}$* mice is increased. (A) Lean mass of WT (black dots) and *Panx1$^{-/-}$* mice (red dots) housed at 22°C. **(B)** Ambulatory, **(C)** fine and **(D)** total activity as well as **(H)** the VO_2 and **(I)** the VCO_2 were measured by indirect calorimetry. Mice were housed two per cage. Each cage was considered as one pooled sample (N=3). **(E)** Representative images of small intestines from WT and *Panx1$^{-/-}$* mice and quantification of small intestine **(F)** and colon (**G**) lengths. Data are shown as individual data points, except for time lines where the number of mice is specified, and expressed as mean ± SEM. $^{†} p \leq 0.01$.

Supplementary figure S3: Cold exposure increases the activity level of 20 weeks-old *Panx1$^{-/-}$* mice.

Body temperatures of WT (in black) and *Panx1$^{-/-}$* mice (in red) during **(A)** the week of acclimatization to 14°C and **(B)** the first 72 hours of exposure to 6°C. **(C)** Lean mass

of WT (black dots) and *Panx1$^{-/-}$* mice (red dots) housed at 6°C. **(D)** Ambulatory, **(E)** fine and **(F)** total activity as well as **(J)** the VO_2 and **(K)** the VCO_2 were measured by indirect calorimetry in WT and *Panx1$^{-/-}$* mice. Mice were housed two per cage. Each cage was considered as one pooled sample (N=3). **(G)** Representative images of small intestines from WT and *Panx1$^{-/-}$* mice and quantification of small intestine **(H)** and colon (**I**) lengths. * $p \leq 0.05$ and $^{\dagger}p \leq 0.01$.
