# Supplementary figures and images for: Cold Exposure Rejuvenates the Metabolic Phenotype of Panx1−/− Mice
Source: Biomolecules. 2024 Aug 25;14(9):1058. doi: 10.3390/biom14091058 (PMC11430693; doi:10.3390/biom14091058)

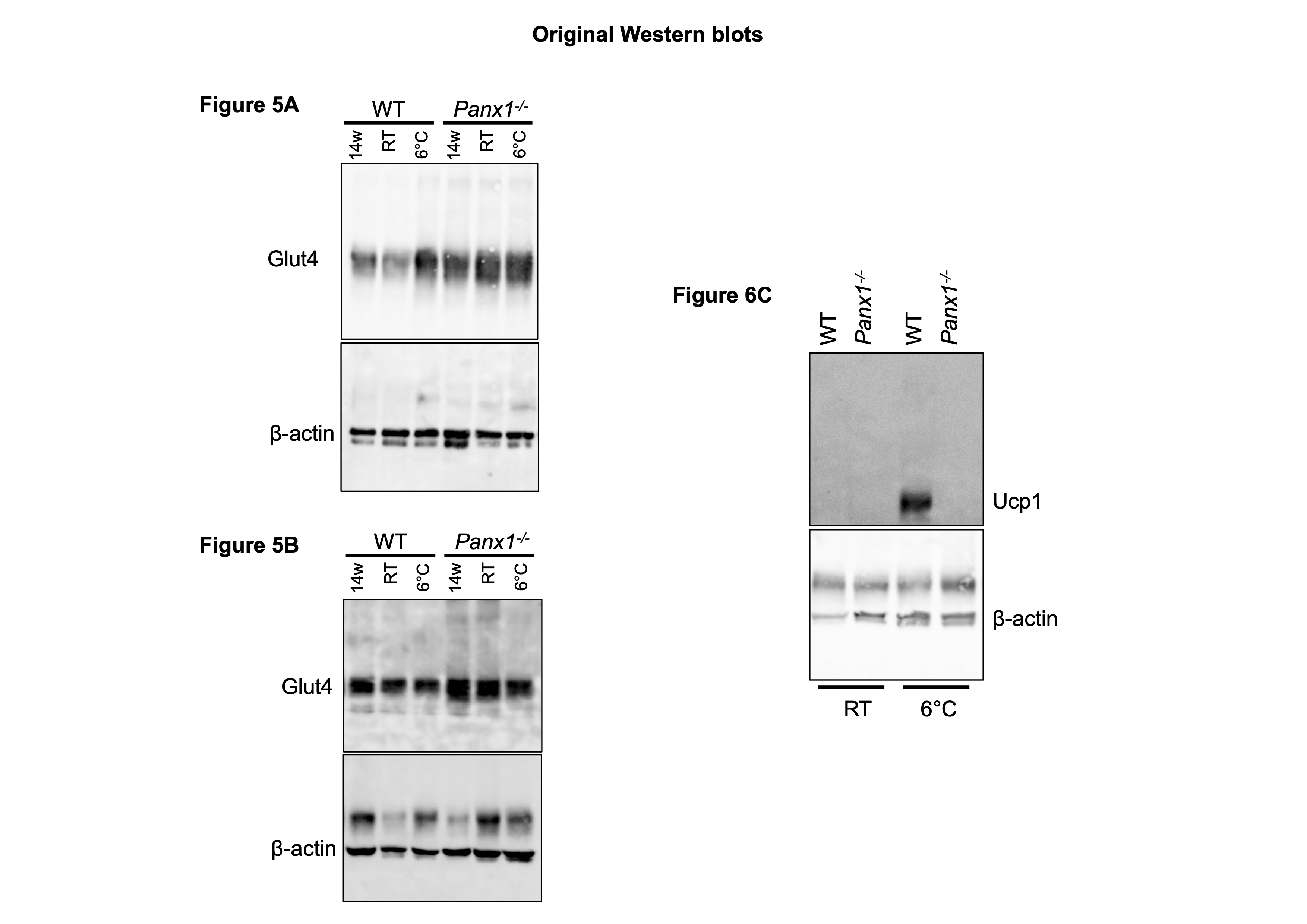

Supplement: Supplementary file 1 [file biomolecules-14-01058-s001.zip › Original Western blots.tiff]

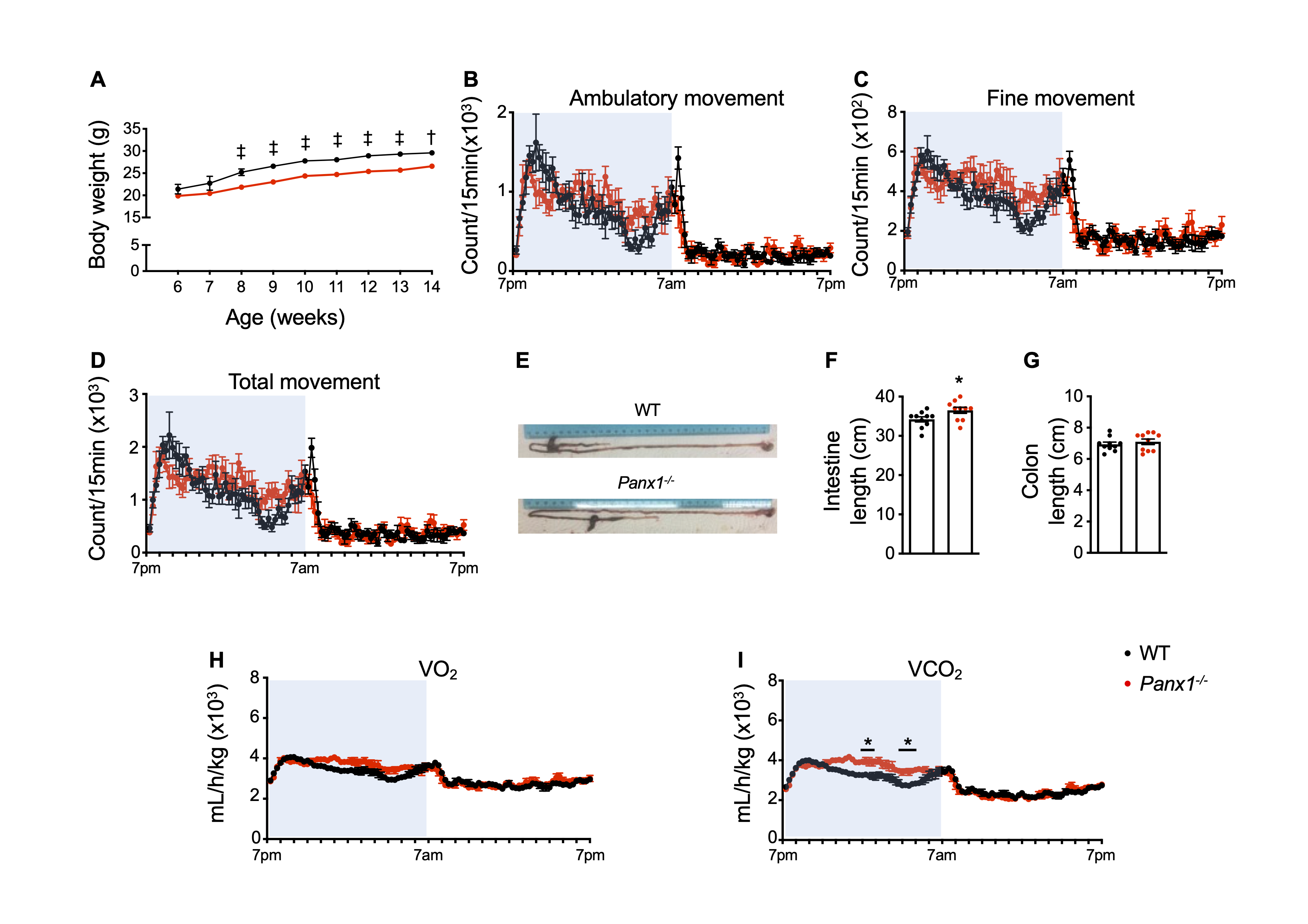

Supplement: Supplementary file 1 [file biomolecules-14-01058-s001.zip › Supp. Figure S1.tiff]

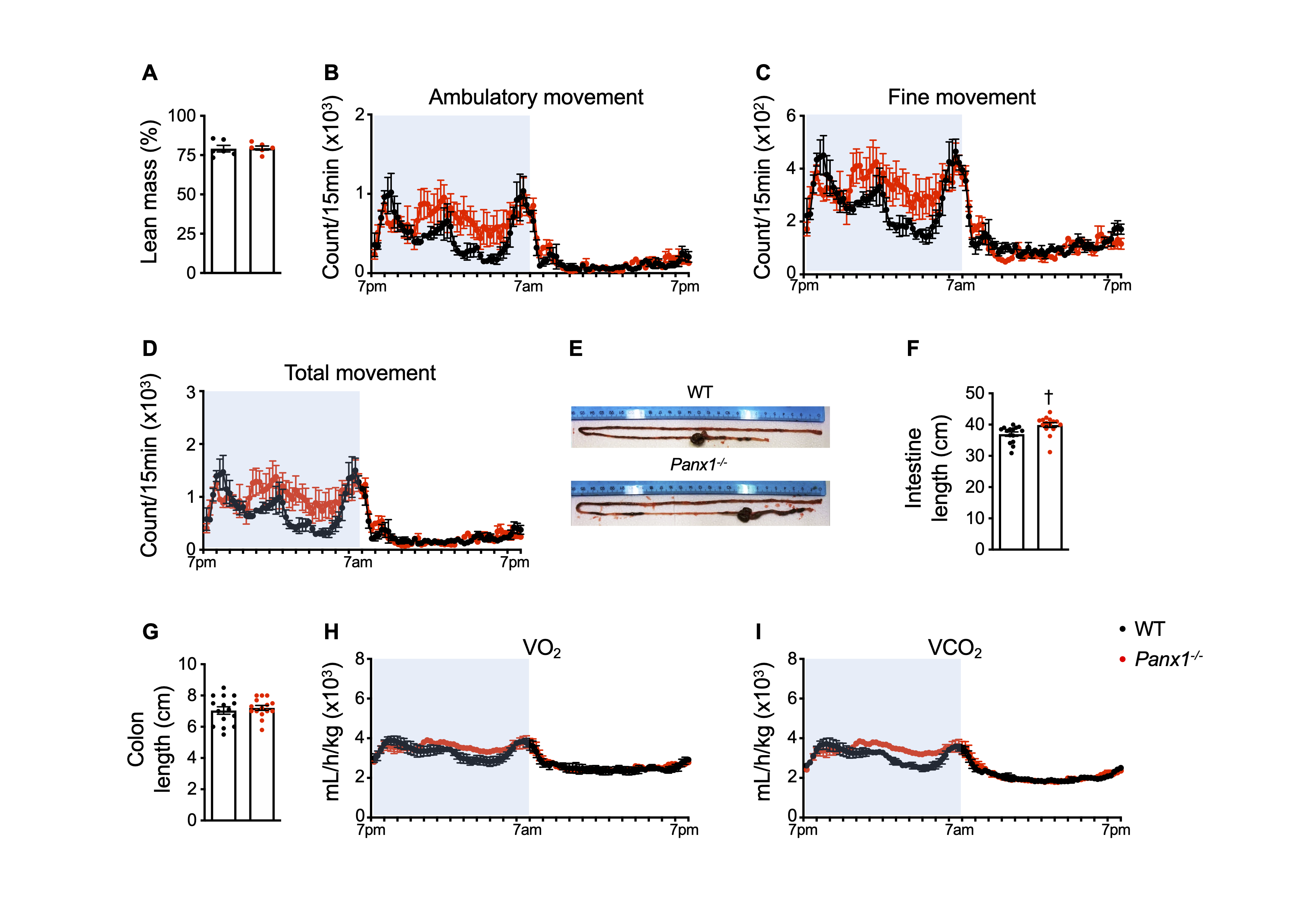

Supplement: Supplementary file 1 [file biomolecules-14-01058-s001.zip › Supp. Figure S2.tiff]

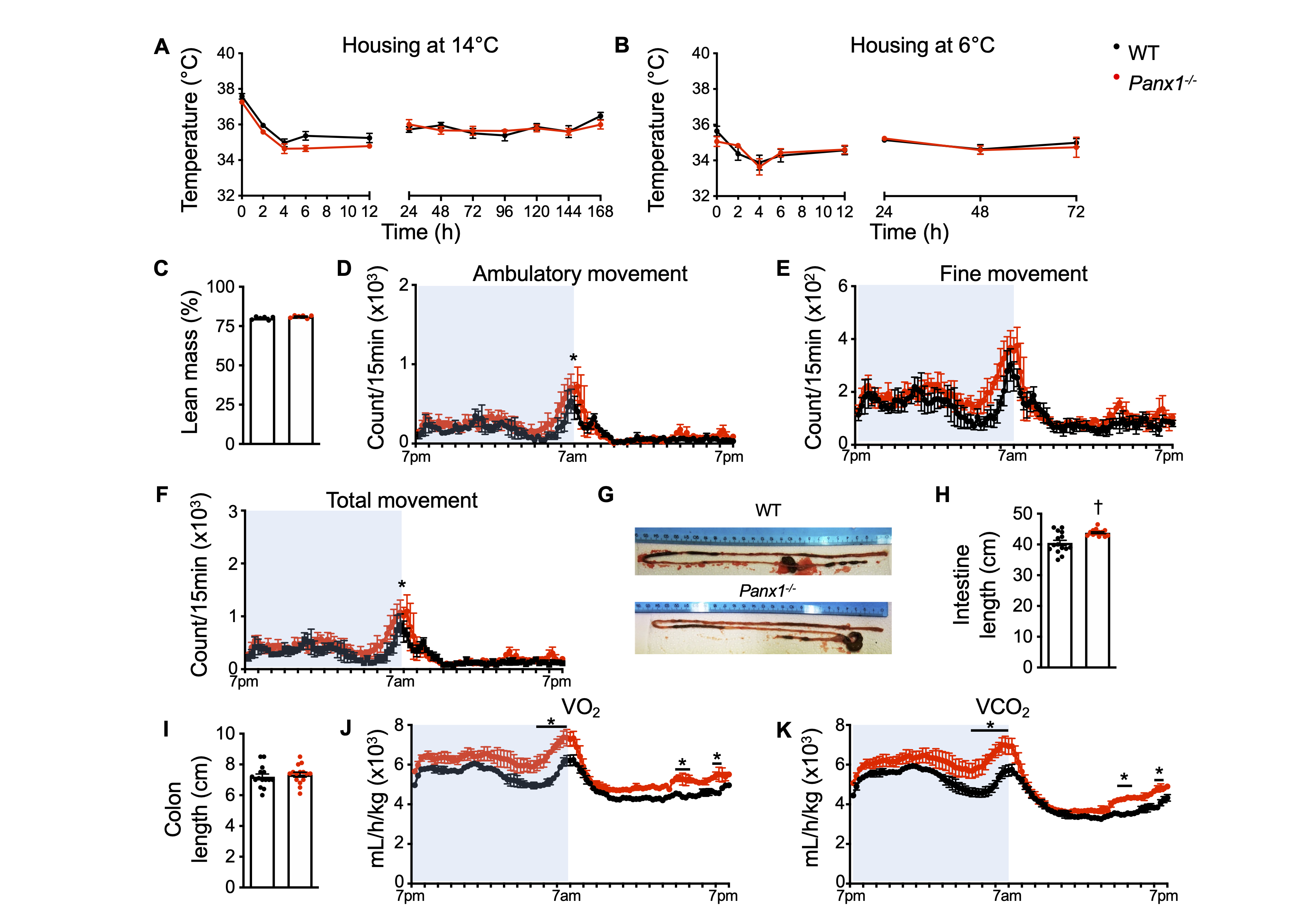

Supplement: Supplementary file 1 [file biomolecules-14-01058-s001.zip › Supp. Figure S3.tiff]
